# Sharpey‐Schafer, Langley and Sherrington: ‘swordsmen’ of physiology. A historical look to the future

**DOI:** 10.1113/EP091172

**Published:** 2023-03-15

**Authors:** Damian M. Bailey, Ronan M. G. Berg, Alex Stewart, Josephine C. Adams, Peter Kohl

**Affiliations:** ^1^ Neurovascular Research Laboratory, Faculty of Life Sciences and Education University of South Wales Pontypridd UK; ^2^ Centre for Physical Activity Research Copenhagen University Hospital – Rigshospitalet Copenhagen Denmark; ^3^ Department of Clinical Physiology and Nuclear Medicine Copenhagen University Hospital – Rigshospitalet Copenhagen Denmark; ^4^ Department of Biomedical Sciences, Faculty of Health and Medical Sciences University of Copenhagen Copenhagen Denmark; ^5^ The Physiological Society London UK; ^6^ School of Biochemistry University of Bristol Bristol UK; ^7^ Institute for Experimental Cardiovascular Medicine, Faculty of Medicine University of Freiburg Freiburg im Breisgau Germany

*Experimental Physiology*, initially entitled *Quarterly Journal of Experimental Physiology*, was established by Sir Edward A. Sharpey‐Schafer (born Edward A. Schäfer, 1850–1935, Figure 1), who was one of the founding members of The Physiological Society (Sharpey‐Schafer, 1927). He remained Chairman of the Editorial Board of *Quarterly Journal of Experimental Physiology* from its inception in 1908 until his retirement 25 years later, in 1933. The first volume was issued at a time when British physiology had taken centre stage internationally, thanks to the foresight of The Physiological Society, which inspired the introduction of physiology schools at most major universities, and the success of *The Journal of Physiology*, which was world‐leading within its specialist field (Johnson, 2021). The birth of *Quarterly Journal of Experimental Physiology* turned out to be a dramatic one, and a cause of considerable tension among members of The Physiological Society (Whitteridge, 1983).

**FIGURE 1:**
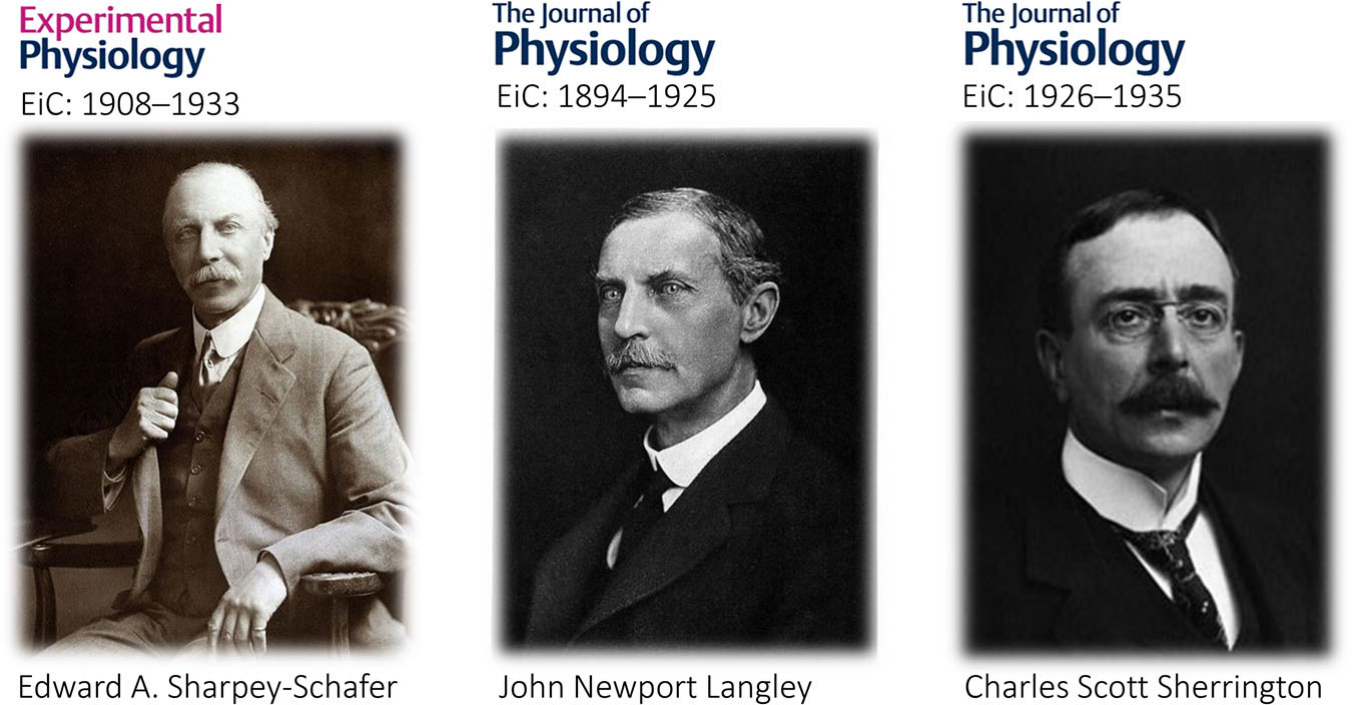
Colorful characters: ‘swordsmen’ of physiology. Images (modified) copyright The Physiological Society (https://cdn.knightlab.com/libs/timeline3/latest/embed/index.html?source=11_DLzCxh0DY_6SWSlMCwgVWSirAhsndlWAgGe1zNkyg&font=Default&lang=en&initial_zoom=2&;height=650). EiC, Editor‐in‐Chief (tenure duration).

As a scientist, Sharpey‐Schafer was a renaissance man, a polymath who made seminal contributions to a variety of fields, ranging from the neuron doctrine (Schäfer, 1878) to the establishment of endocrinology (Schäfer, 1916), discovery of the pressor effects of adrenaline (Oliver & Schäfer, 1895) and development of the ‘Schäfer method’ of artificial respiration (Schäfer, 1903a). The latter stands as testament to his practical approach to physiology, as it was adopted by the Royal Life Saving Society, becoming the standard method for life saving until it was eventually replaced by mouth‐to‐mouth resuscitation decades later. Throughout his career as an experimentalist, teacher and editor, he remained a passionate exponent of experimentally based physiology, which had emerged in Germany and France in the 19th century as a prerequisite for the practice of clinical medicine (Schäfer, 1885, 1903b). This approach had fundamentally liberated physiology from the teleologically based reasoning of natural philosophy, which had dominated the field during its early history (Johnson, 2021). Hence, from being a theoretically based discourse, that is, the ‘speculative wing of anatomy’ that relied exclusively on ‘the pen’, physiology shifted to an experimental discipline of ‘the sword’, whereby the functions of organs and their interplay were studied directly by cutting into living organisms and introducing various perturbations to unveil fundamental homeostatic mechanisms (Cunningham, 2002). This was based on systematic methodology, notably vivisection, as well as critical analysis and formal data presentation that incorporated the ‘graphical method’, that is, providing the reader with illustrations of experimental set‐ups and readouts of representative experimental data.

Before he founded *Quarterly Journal of Experimental Physiology*, Sharpey‐Schafer served on the Editorial Board of *The Journal of Physiology* from 1893, but he was unable to exert much influence due to the constraints imposed by John Newport Langley (1852–1925, Figure 1). Langley bought the journal from its founder, Sir Michael Foster (1836–1907), in 1894 to pay off its considerable debts. Langley and Foster then appeared as co‐Chairs of the Editorial Board on the cover of the journal (Sharpey‐Schafer, 1927), but in practice, it was Langley who took sole ownership of running the journal, responsible for administration, finances, press management, correspondence and editing of papers. He communicated his notorious editorial philosophy in his Presidential address to the Physiological Section of the British Association for the Advancement of Science in 1899: ‘Much that he [a scientist] is forced to read consists of records of defective experiments, confused statements of results, wearisome description of detail, and unnecessarily protracted discussion of unnecessary hypotheses. The publication of such matter is a serious injury to the man of science’ (Fletcher, 1926).

Langley saw himself at war with poor scientific conduct and redundant writing. In his efforts to ensure that a paper was cast in the most effective form, he would often rewrite papers from scratch, regardless of whether the author was a novice or established scientist. This would typically involve shortening the paper substantially and omitting almost all discussion of data and figures, as he insisted that facts must speak for themselves (Fletcher, 1926). Langley's vision and practice for publishing physiology research did not resonate with his colleagues within The Physiological Society, as many saw this as an attack on their academic freedom. Indeed, some bitterly resented the process of having a paper edited under Langley's strict hand, an experience to which they referred as being ‘Langleyized’ (Whitteridge, 1983), a word originally coined by Alexander Forbes (1862–1965) in 1922 (Hodgkin, 1979).

When Foster died in 1907, Langley was suddenly thrust into a position of almost unlimited power as he became the singular editor of *The Journal of Physiology*, which was now the only British physiology journal, given that the only alternative outlet, *The Journal of Anatomy and Physiology*, had disbanded a year prior. A revolt was in the making, and all eyes turned towards Sharpey‐Schafer, who had tried, albeit unsuccessfully, to revive the latter journal. Like many others, Sharpey‐Schafer found Langley's autocratic editorial style worrisome, and he was also sceptical of his unwillingness to go to the expense of publishing figures (Whitteridge, 1983). Indeed, Sharpey‐Schafer's editorial philosophy was the polar opposite of Langley's; referring to authors of submitted papers, he would laconically say (Sherrington, 1935): ‘Their readers are their judges, not I’.

However, it was Sharpey‐Schafer's friend, Sir Charles Scott Sherrington (1857–1952, Figure 1), who was responsible for encouraging him to start up an entirely ‘new’ physiology journal. Sherrington had previously written under Sharpey‐Schafer's editorship when he contributed four chapters on neurophysiology for the acclaimed and widely read *Text‐book of Physiology* published a decade earlier (Schäfer, 1900). He had trained and conducted his first physiology experiments under Langley's supervision in the early 1880s. By 1907, Sherrington was an established independent investigator. In his lifetime, he received no less than 131 Nobel Prize nominations: a prize he was eventually (jointly) awarded together with Edgar Douglas Adrian (1889–1977) in 1932 for ground‐breaking work on the function of neurons. Yet despite his contributions, he continually suffered under the strict editorial policy of his former tutor, seeing one paper after another being ‘Langleyized’ before eventual publication in *The Journal of Physiology*.

Before studying physiology, Sherrington was a classics scholar with an arts degree, and an enthusiastic poet. This creativity was evident in his unique scientific writing style, described as consisting of ‘pregnant, intricate sentences with sudden inversions or flashes of imagery to drive home his meaning’ (Adrian, 1952). This approach conflicted with Langley's views on how to present and discuss scientific data. Indeed, his first paper submitted to *The Journal of Physiology* after Langley had become co‐Chairman of the Editorial Board, a landmark study on the sensory function of muscle spindles (Sherrington, 1894), included many carefully prepared histological illustrations, which to his disappointment, were all immediately discarded by Langley (Eccles & Gibson, 2012). It was Sherrington who wrote a letter to Sharpey‐Schafer in June 1907, less than 6 months after Foster's death, stating: ‘A new Journal is wanted and you are the person to start it!’ This served as the catalyst for the inception of *Quarterly Journal of Experimental Physiology* (Whitteridge, 1983).

Soon, Sharpey‐Schafer found himself on a quest to start a new physiology journal with collective support from members of The Physiological Society. The new journal would accommodate an increasing demand for scientific journals published in English due to the popularity of physiology in Great Britain and in the face of no less than four competitor German journals: *Archiv für Physiologie*, *Zentralblatt für Physiologie*, *Archiv für Anatomie und Physiologie* and *Archiv für die gesamte* *Physiologie des Menschen und der Tiere* (amalgamated and later renamed to become *Pflüger's Archiv für die gesamte Physiologie des Menschen und der Tiere*). But Sharpey‐Schafer's main motivation was to publish papers in the original form submitted by the author, making full use of illustrations, challenging Langley's editorial philosophy originally published in *The Journal of Physiology* (Sherrington, 1935).

*The Journal of Physiology* and *Experimental Physiology* subsequently evolved as the two main publication outlets for international physiological research in Great Britain. Both were eventually assimilated as official journals of The Physiological Society, *The Journal of Physiology* in 1926 after Langley's untimely death, and *Quarterly Journal of Experimental Physiology* half a century later in 1979 when it narrowly survived near‐bankruptcy. Under the auspices of *The Physiological Society*, they were joined in 2013 by a third ‘sister journal’ – *Physiological Reports*, an inclusive, open‐access, online journal co‐owned by the American Physiological Society, and started under founding Editor Sue Wray. With its emphasis on sound science, rather than the perceived novelty or completeness of an advance made, *Physiological Reports* also offers authors the possibility to publish research findings that are incompletely mechanistic, correlative, have negative outcomes, or are overlapping with or confirmatory of prior work (Adams, 2022). In addition to direct submissions, many manuscripts are transferred by authors to *Physiological Reports* after consideration at other (The Physiological Society or American Physiological Society) journals, accompanied by any peer‐review reports. This provides an expedited route to final publication and increases the overall efficiency of the peer‐review system. While some to this day still claim that *The Journal of Physiology* and *Experimental Physiology* are ‘sisters with issues’ harking back to colourful days of old, this no longer holds true. Although the inception of *Experimental Physiology* was clearly a rebellious act, science and publishing policies have evolved considerably over the past 115 years, one would hope! To be fair, it was Langley who was admittedly ahead of his time, demanding a rigorously structured approach to the analysis and presentation of data. Apart from his strong aversion to the ‘graphical method’, Langley's philosophy prevailed, even when Sherrington superseded him as Chairman of the Editorial Board of *The Journal of Physiology* (although the Editorial Board admittedly did become much more involved under Sherrington's leadership) and has deeply influenced how scientific papers are written across all life sciences to this day. Eventually, both Sharpey‐Schafer and Sherrington, among others, recognised without reservation that much of the success of *The Journal of Physiology* and of British physiology in general was down to Langley's efforts as an editor (Adrian, 1952; Fletcher, 1926; Sharpey‐schafer, 1927; Sherrington, 1926). Despite markedly different approaches to scientific writing and publishing, Sharpey‐Schafer, Sherrington and Langley were all ‘swordsmen’ fighting on the same side, barking up the same tree, pushing to promote and develop experimentally based physiology.

In modern times, physiology remains an inherently experimental science closely linked to clinical medicine, a truth reflected in *Experimental Physiology*’s title and translational scope. The title of the journal has undergone several iterations during its lifetime; between 1938 and 1980 it was entitled *Quarterly Journal of Experimental Physiology and Cognate Medical Sciences* to stress these links. Between 1981 and 1989, it was changed back to *Quarterly Journal of Experimental Physiology*, but since it was no longer a quarterly journal, the title was shortened to the current *Experimental Physiology* in 1990. Surely, the exclusion of redundant words in the title would have pleased the late Langley, and with its focus on integrative translational physiology, it still aligns with Sharpey‐Schafer's original vision, for both the journal and wider field of physiology.

But perhaps most importantly of all, *The Journal of Physiology* and *Experimental Physiology* should no longer be viewed as ‘competitive rivals’ or ‘sisters with issues’. To the contrary, we now view them, jointly with *Physiological Reports*, as ‘synergistic sisters’, representing independent yet complementary brands that collectively serve the wider community by promoting the ‘power of physiology’ in a modern and more inclusive manner (Adams, 2022; Bailey, 2022; Bailey & Stewart, 2022; Kohl, 2022). Since the founding of *The Journal of Physiology* and *Experimental Physiology*, hundreds of physiology journals have become established around the world. Given the increasingly competitive publishing landscape, we have every reason to look for the synergies between our sisterhood of three publications, and rely on each other, as we look to set the bar high for the rest of the field. Examples of our collaborative synergies include the Arrive 2.0 guidelines (joint animal and human experimental policies), joint statistical reporting policies, and an increased focus on transfer of articles between the three journals, where possible. With the support of talented staff in our respective publishing offices, more initiatives between the three journals are planned as the current Editors‐in‐Chief look to forge a future of mutual respect and benefit.

## AUTHOR CONTRIBUTIONS

Damian M. Bailey conceived the idea following discussion with Peter Kohl and wrote the first draft of the manuscript with Ronan M. G. Berg. Damian M. Bailey, Ronan M. G. Berg, Alex Stewart, Josephine C. Adams and Peter Kohl edited and revised the manuscript. Damian M. Bailey, Ronan M. G. Berg, Alex Stewart, Josephine C. Adams and Peter Kohl approved the final version submitted for publication and agree to be accountable for all aspects of the work in ensuring that questions related to the accuracy or integrity of any part of the work are appropriately investigated and resolved. All persons designated as authors qualify for authorship, and all those who qualify for authorship are listed.

## CONFLICT OF INTEREST

None declared.

## FUNDING INFORMATION

None.
